# Frailty index based on laboratory tests improves prediction of short-and long-term mortality in patients with critical acute myocardial infarction

**DOI:** 10.3389/fmed.2022.1070951

**Published:** 2022-12-06

**Authors:** Weimin Bai, Benchuan Hao, Lijun Xu, Ji Qin, Weihao Xu, Lijie Qin

**Affiliations:** ^1^Department of Emergency, Henan Provincial People’s Hospital, People’s Hospital of Zhengzhou University, People’s Hospital of Henan University, Zhengzhou, China; ^2^Medical School of Chinese PLA, Beijing, China; ^3^Department of Cardiology, The Second Medical Center and National Clinical Research Center for Geriatric Diseases, Chinese PLA General Hospital, Beijing, China; ^4^Haikou Cadre’s Sanitarium of Hainan Military Region, Haikou, China

**Keywords:** frailty, frailty index, laboratory tests, acute myocardial infarction, mortality

## Abstract

**Background:**

Previous studies have shown that the frailty index based on laboratory tests (FI-Lab) can identify older adults at increased risk of adverse health outcomes. This study aimed to determine whether the FI-Lab is associated with mortality risk and can provide incremental improvements in risk stratification of patients with critical acute myocardial infarction (AMI).

**Materials and methods:**

We conducted a secondary analysis of data from the Medical Information Mart for Intensive Care (MIMIC)-IV database. A 33-item FI-Lab was constructed. Outcomes of interest were in-hospital and 1-year mortality. Logistic regression models were used to investigate the association between the FI-Lab and outcomes. For the assessment of the incremental predictive value, the FI-Lab was added to several risk stratification scoring systems for critically ill patients, and the following indices were calculated: Δ C-statistic, integrated discrimination improvement (IDI), and net reclassification improvement (NRI).

**Results:**

Out of 2,159 patients, 477 died in hospital (22.1%), and 898 died during the 1-year follow-up period. After adjustment for confounders, the FI-Lab was associated with increased in-hospital mortality [odds ratio (OR) = 1.06, 95% confidence interval (CI): 1.05–1.07] and 1-year mortality (OR = 1.05, 95% CI: 1.04–1.06) when assessed as a continuous variable (per 0.01-score increase). When assessed as a categorical variable, the FI-Lab was associated with in-hospital mortality (2nd Quartile: OR = 1.89, 95% CI: 1.18–3.03; 3rd Quartile: OR = 3.46, 95% CI: 2.20–5.46; and 4th Quartile: OR = 5.79, 95% CI: 3.61–9.28 compared to 1st Quartile) as well as 1-year mortality (2nd Quartile: OR = 1.66, 95% CI: 1.23–2.24; 3rd Quartile: OR = 2.40, 95% CI: 1.76–3.26; and 4th Quartile: OR = 3.76, 95% CI: 2.66–5.30 compared to 1st Quartile) after adjustment for confounders. The addition of the FI-Lab to all disease severity scores improved discrimination and significantly reclassified in-hospital and 1-year mortality risk.

**Conclusion:**

The FI-Lab was a strong predictor of short- and long-term mortality in patients with critical AMI. The FI-Lab improved the ability to predict mortality in patients with critical AMI and therefore might be useful in the clinical decision-making process.

## Background

Acute myocardial infarction (AMI) is an acute and critical illness that is encountered in clinical practice. Globally, AMI has been a public health challenge in recent years. According to the latest heart disease statistics of the US, the overall prevalence of MI is 3.0% in US adults ≥ 20 years of age (1). The estimated annual incidence of MI is 605,000 new attacks and 200,000 recurrent attacks (1). The prognosis of AMI patients is dismal, especially for those with ST-segment elevation myocardial infarction (STEMI). Data showed that 110,346 patients died from AMI in 2017 in the US (1). In China, the estimated mortality rate of AMI was 60.20 per 100,000 for urban areas and 78.24 per 100,000 for rural areas (2). The burden caused by AMI is very high and continues to increase. Identifying new risk factors to perform effective interventions is essential for the clinical management of AMI.

Frailty is a syndrome that is characterized by decreased reserves of biological systems (3). Frail patients are vulnerable to internal or external stimuli and have higher risk of various adverse events, including disability and mortality (4). Recently, frailty has been recognized as an important prognostic indicator for AMI (5–7). Routine frailty assessment in the clinical management of AMI can identify a high-risk subgroup of patients with a poor prognosis. A previous meta-analysis showed that several frailty assessment tools have been used in AMI patients, including the Fried phenotype, the frailty index (FI), the FRAIL scale, the Edmonton frail scale (EFS), the Green score, and the clinical frailty scale (CFS) (8). The FI based on laboratory tests (FI-Lab) was first proposed by Howlett et al. and can be easily constructed in routine clinical practice (9). Subsequent research based on community populations and hospitalized patients has confirmed the predictive ability of the FI-Lab for a wide range of health outcomes, including mortality, institutionalization, increased medication use, increased number of physician visits, and decreased self-rated health (10–14). However, the FI-Lab has not yet been applied to AMI patients.

In the current study, we analyzed data from the Medical Information Mart for Intensive Care (MIMIC)-IV database to examine the association between the FI-Lab and in-hospital and 1-year mortality among critical AMI patients. Furthermore, we investigated the incremental value of adding the FI-Lab to disease severity scoring systems, including sequential organ failure assessment (SOFA), acute physiology score (APS) 3, simplified acute physiology score (SAPS) 2, logistic organ dysfunction score (LODS), Oxford acute severity of illness score (OASIS), and systemic inflammatory response syndrome score (SIRS).

## Materials and methods

### Study design and population

This retrospective study used data from the MIMIC-IV database (certification number: 10713670). The MIMIC-IV database contains 454,324 hospital admission records and 76,943 intensive care unit (ICU) admission records of patients admitted to the Beth Israel Deaconess Medical Center (BIDMC, Boston, MA, USA) from 2008–2019. Details have been provided in previous literature (15, 16).

Patients who were diagnosed with AMI and had sufficient data to calculate the FI-Lab were eligible for this study. The following ICD codes were used to identify and extract patients with AMI in the MIMIC-IV database: [1] ICD-9: 410.0 (410.00, 410.01, and 410.02), 410.1 (410.10, 410.11, and 410.12), 410.2 (410.20, 410.21, and 410.22), 410.3 (410.30, 410.31, and 410.32), 410.4 (410.40, 410.41, and 410.42), 410.5 (410.50, 410.51, and 410.52), 410.6 (410.60, 410.61, and 410.62), 410.7 (410.70, 410.71, and 410.72), 410.8 (410.80, 410.81, and 410.82), and 410.9 (410.90, 410.91, and 410.92); and [2] ICD-10: I21.0 (I21.01, I21.02, and I21.09), I21.1 (I21.11 and I21.19), I21.2 (I21.21 and I21.29), I21.3, and I21.4.

The exclusion criteria included: (1) patients under 18 years old; (2) patients with a survival time < 24 h; (3) organ donors; (4) patients who were pregnant, had recently given birth, or had puerperal illness; and (5) patients with missing key variables (e.g., demographic data, SOFA, APS 3, SAPS 2, LODS, OASIS, and SIRS) for analyses. If patients with AMI had more than one ICU admission record, only the first ICU admission record was included in the analysis.

### Frailty index based on laboratory tests

A total of 33 items were used to construct the FI-Lab, including 30 laboratory test items (obtained during the 24 h before and after ICU admission) from venous blood samples (white cell count, platelet count, hemoglobin, total bilirubin, alanine transaminase, albumin, alkaline phosphatase, lactate dehydrogenase, urea nitrogen, creatinine, glucose, potassium, sodium, calcium, phosphorus, prothrombin time, international normalized ratio, activated partial thromboplastin time, fibrinogen, and Troponin T), arterial blood gas samples (potential of hydrogen, partial pressure of oxygen, partial pressure of carbon dioxide, and lactate), urine samples (leucocytes, erythrocytes, protein, glucose, ketones, and bilirubin), and three vital signs: systolic blood pressure, diastolic blood pressure, and heart rate. We dichotomized each item using the normal reference intervals provided in the database; any value outside of the reference range was given the score of 1 as a deficit. The reference value of each items are presented in Supplementary Table 1. In the present study, the FI-Lab score was calculated by summing the deficits present and then dividing the summed amount by the number of items included. The FI-Lab theoretically ranges from 0 to 1. Only patients who possessed more than 80% of the necessary items (*n* = 27) were included. The FI-Lab levels were categorized by quartiles.

### Covariates

Covariate variables included age, gender, ethnicity (White, Black, and other), AMI type (STEMI and non-STEMI), troponin T level, and disease severity scores (SOFA, APS3, SAPS2, LODS, OASIS, and SIRS). The detailed scoring rules of the disease severity scores were shown in Supplementary Tables 2–7.

### Outcomes

Outcomes of interest of this study were in-hospital and 1-year mortality.

### Statistical analysis

We described and compared the baseline characteristics of the study population based on their different FI-Lab levels (1st Quartile, 2nd Quartile, 3rd Quartile: OR, and 4th Quartile). Continuous variables were described as the median (25% quartile, 75% quartile) owing to non-normal distributions, while categorical variables were described as frequencies and percentages. We used the Kruskal–Wallis *H* test to compare the differences in continuous variables and a χ^2^ test to compare differences in categorical variables between groups. We applied logistics regression to investigate the association between the FI-Lab (as a continuous or categorical variable) and in-hospital and 1-year mortality. Age, gender, ethnicity, AMI type, troponin T level, and the SOFA scores were adjusted in the multivariable model to determine the association between the FI-Lab and mortality. We used the metrics of discrimination (Harrel’s C statistic) to assess the model’s predictive performance, and the DeLong test was used to compare the C statistics of model including and not including FI-Lab. We calculated the Δ C-statistic, integrated discrimination improvement (IDI), and net reclassification improvement (NRI) to determine the incremental predictive value of adding the FI-lab (as a continuous variable) to the base model for in-hospital and 1-year mortality (17). We conducted statistical analyses using SPSS 26.0 for Windows (SPSS Inc., Chicago, IL, USA) and R (version 4.1.2). *P* < 0.05 was considered statistically significant.

## Results

### Characteristics of the study population

A total of 2,159 critical patients were included in the analyses. Supplementary Figure 1 shows the detailed selection process. No significant difference in baseline clinical parameters was present between included and excluded patients except lower troponin T level (median, 0.40 ng/mL vs. 0.48 ng/mL) (Supplementary Table 8). The baseline characteristics of the included patients are presented in Table 1. The median age was 72 (62–81) years. Men accounted for 60.1% (*n* = 1,298) of the participants, and the median FI-Lab was 0.45 (0.36–0.55) points. Patients with higher FI-Lab scores were older and had higher levels of all disease severity scores (SOFA, APS 3, SAPS 2, LODS, OASIS, and SIRS); additionally, a higher proportion of them had a diagnosis of STEMI.

**TABLE 1:** Baseline characteristics of included patients according to frailty index based on laboratory tests (FI-Lab) levels (*n* = 2,159).

	FI-Lab categories				
	
Variable	Quartile 1 (*n* = 542)	Quartile 2 (*n* = 585)	Quartile 3 (*n* = 543)	Quartile 4 (*n* = 489)	*P*-value
Age (year)	70 (61–80)	71 (61–80)	73 (64–81)	74 (63–82)	0.003
Men (n, %)	308 (56.8%)	346 (59.1%)	343 (63.2%)	301 (61.6%)	0.157
**Ethnicity (n, %)**					0.156
White	352 (64.9%)	382 (65.3%)	352 (64.8%)	286 (58.5%)	
Black	38 (7.0%)	50 (8.5%)	37 (6.8%)	48 (9.8%)	
Other	152 (28%)	153 (26.2%)	154 (28.4%)	155 (31.7%)	
STEMI (n, %)	160 (29.5%)	185 (8.6%)	202 (37.2%)	197 (40.3%)	0.001
Troponin T (ng/mL)	0.26 (0.08–0.77)	0.40 (0.12–1.33)	0.43 (0.15–1.61)	0.57 (0.17–2.36)	<0.001
FI-Lab (score)	0.33 (0.27–0.36)	0.42 (0.39–0.45)	0.51 (0.48–0.54)	0.61 (0.58–0.66)	<0.001
**Disease severity scoring system (score)**					
SOFA	4 (2–6)	5 (3–7)	6 (4–9)	9 (6–11)	<0.001
APS 3	37 (28–49)	48 (38–60)	58 (46–73)	77 (60–102)	<0.001
SAPS 2	34 (28–41)	38 (32–45)	44 (37–52)	52 (43–65)	<0.001
LODS	4 (2–6)	5 (3–7)	7 (5–9)	9 (6–12)	<0.001
OASIS	31 (26–38)	33 (27–40)	37 (31–43)	42 (35–50)	<0.001
SIRS	2 (2–3)	3 (2–3)	3 (2–3)	3 (3–4)	<0.001
In-hospital death (n, %)	31 (5.7%)	79 (13.5%)	142 (26.2%)	225 (46.0%)	<0.001
1-year death (n, %)	112 (20.7%)	214 (36.6%)	262 (48.3%)	310 (63.4%)	<0.001

FI-Lab, frailty index based on laboratory tests; STEMI, ST-segment elevation myocardial infarction; SOFA, sequential organ failure assessment; APS, acute physiology score; SAPS, simplified acute physiology score; LODS, logistic organ dysfunction score; OASIS, Oxford acute severity of illness score; SIRS, systemic inflammatory response syndrome.

### Association between frailty and in-hospital and 1-year mortality

**FIGURE 1:**
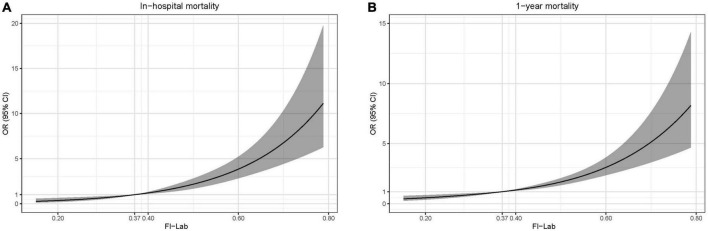
Spline curves showing the association of the frailty index based on laboratory tests (FI-Lab) as a continuous variable with in-hospital mortality **(A)** and 1-year mortality **(B)** the dotted line (0.37) indicates the 25% quantile of the FI-Lab distribution. Spline curves were adjusted for age, gender, ethnicity, acute myocardial infarction (AMI) type, and sequential organ failure assessment (SOFA) score.

A total of 477 patients died in the hospital (22.1%). The mortality rate increased significantly with the elevation of the FI-Lab (Table 1). The multivariate model showed that age, gender, ethnicity (White), troponin T level, the FI-Lab, and all disease severity scores (SOFA, APS 3, SAPS 2, LODS, OASIS, and SIRS) were independent risk factors of in-hospital mortality (Table 2). The FI-Lab was associated with increased in-hospital mortality both as a continuous variable [per 0.01-score increase: odds ratio (OR) = 1.06, 95% confidential interval (CI) 1.05–1.07] and as a categorical variable (OR = 1.89, 95% CI: 1.18–3.03 for the 2nd quartile, OR = 3.46, 95% CI: 2.20–5.46 for the 3rd quartile, and OR = 5.79, 95% CI: 3.61–9.28 for the 4th quartile) in the multivariable model (Figure 1; Table 3). There was a significant trend of increasing cumulative odds of in-hospital mortality with a higher FI-Lab level (*P* for trend < 0.001). The univariate OR and C-statistic for in-hospital mortality of each items included in the FI-Lab were shown in Supplementary Table 9.

**TABLE 2:** Association of baseline characteristics with in-hospital and 1-year mortality.

Variables	In-hospital mortality		1-year mortality	
		
	Univariate model	Multivariable model	Univariate model	Multivariable model
Age (per 5 years)	1.09 (1.05–1.13)	1.15 (1.10–1.21)	1.22 (1.17–1.26)	1.27 (1.22–1.32)
Gender (Men vs. Women)	0.94 (0.68–1.03)	0.74 (0.58–0.94)	0.78 (0.65–0.93)	0.77 (0.63–0.94)
**Ethnicity**				
Other	1.00	1.00	1.00	1.00
White	0.63 (0.50–0.79)	0.65 (0.50–0.85)	0.97 (0.80–1.18)	0.95 (0.76–1.19)
Black	0.73 (0.49–1.09)	0.85 (0.53–1.36)	0.86 (0.61–1.22)	1.06 (0.71–1.60)
STEMI	1.36 (1.11–1.68)	1.05 (0.82–1.35)	1.25 (1.05–1.50)	1.05 (0.85–1.29)
Troponin T	1.11 (1.07–1.14)	1.07 (1.03–1.10)	1.07 (1.04–1.10)	1.05 (1.01–1.08)
FI-Lab (per 0.01-score)	1.09 (1.08–1.10)	1.06 (1.05–1.07)	1.06 (1.05–1.07)	1.05 (1.04–1.06)
**Disease severity scoring system**				
SOFA	1.26 (1.22–1.30)	1.15 (1.11–1.20)	1.16 (1.13–1.19)	1.08 (1.04–1.12)
APS 3	1.04 (1.03–1.05)	1.03 (1.03–1.04)	1.03 (1.03–1.04)	1.02 (1.02–1.03)
SAPS 2	1.07 (1.06–1.08)	1.04 (1.03–1.05)	1.06 (1.05–1.07)	1.03 (1.02–1.04)
LODS	1.34 (1.30–1.39)	1.24 (1.20–1.30)	1.24 (1.30–1.27)	1.17 (1.13–1.21)
OASIS	1.10 (1.09–1.11)	1.07 (1.05–1.08)	1.07 (1.06–1.08)	1.04 (1.03–1.05)
SIRS	1.51 (1.34–1.71)	1.17 (1.01–1.35)	1.22 (1.10–1.35)	1.02 (0.91–1.15)

STEMI, ST-segment elevation myocardial infarction; FI-Lab, frailty index based on laboratory tests; SOFA, sequential organ failure assessment; APS, acute physiology score; SAPS, simplified acute physiology score; LODS, logistic organ dysfunction score; OASIS, Oxford acute severity of illness score; SIRS, systemic inflammatory response syndrome.

**TABLE 3:** Association of frailty index based on laboratory tests (FI-Lab) with in-hospital and 1-year mortality.

	Crude model	Model 1	Model 2
**In-hospital mortality**			
Continuous variable (per 0.01-score)	1.09 (1.08–1.10)	1.09 (1.08–1.10)	1.06 (1.05–1.07)
**Categorical variable**			
Quartile 1	1.00	1.00	1.00
Quartile 2	2.57 (1.67–3.97)	2.50 (1.62–3.86)	1.89 (1.18–3.03)
Quartile 3	5.84 (3.87–8.80)	5.81 (3.85–8.77)	3.46 (2.20–5.46)
Quartile 4	14.05 (9.38–21.04)	14.63 (9.74–21.97)	5.79 (3.61–9.28)
*p* for trend	<0.001	< 0.001	<0.001
**1-year mortality**			
Continuous variable (per 0.01-score)	1.06 (1.05–1.07)	1.07 (1.06–1.08)	1.05 (1.04–1.06)
**Categorical variable**			
Quartile 1	1.00	1.00	1.00
Quartile 2	2.22 (1.70–2.89)	2.13 (1.62–2.80)	1.66 (1.23–2.24)
Quartile 3	3.58 (2.74–4.68)	3.63 (2.75–4.79)	2.40 (1.76–3.26)
Quartile 4	6.65 (5.04–8.78)	7.59 (5.68–10.14)	3.76 (2.66–5.30)
*p* for trend	<0.001	< 0.001	<0.001

FI-Lab, frailty index based on laboratory tests.

Model 1 adjusted for age and sex.

Model 2 adjusted for age, sex, ethnicity, AMI type, troponin T, and SOFA score.

A total of 898 patients died within 1 year (41.6%). The 1-year mortality rate increased significantly with the elevation of the FI-Lab (Table 1). Age, gender, troponin T level, the FI-Lab, and all disease severity scores with the exception of AMI type (STEMI) and SIRS were significantly associated with increased risk of 1-year mortality (Table 2). After adjustment for multiple confounders, the FI-Lab was independently associated with increased 1-year mortality both as a continuous variable (per 0.01-score increase: OR = 1.05, 95% CI: 1.04–1.06) and as a categorical variable (OR = 1.66, 95% CI: 1.23–2.24 for the 2nd quartile, OR = 2.40, 95% CI: 1.76–3.26 for the 3rd quartile, and OR = 3.76, 95% CI: 2.66–5.308 for the 4th quartile) (Figure 1; Table 3). There was also a significant trend of increasing cumulative odds of 1-year mortality with a higher FI-Lab level (*P* for trend < 0.001). The univariate OR and C-statistic for 1-year mortality of each items included in the FI-Lab were shown in Supplementary Table 10.

### Incremental value of adding the frailty index based on laboratory to disease severity scores for mortality

**FIGURE 2:**
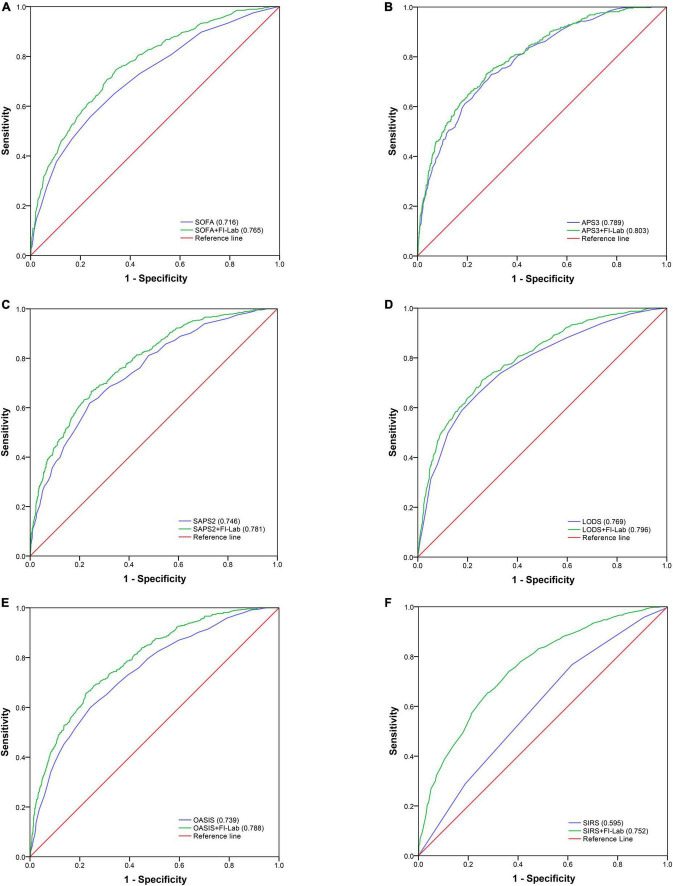
Area under the receiver operator curve for in-hospital mortality.

Sequential organ failure assessment (SOFA), APS 3, SAPS 2, LODS, and OASIS all had good discrimination ability for in-hospital mortality [area under the receiver operating characteristic curve (AUROC) = 0.716, 0.789, 0.746, 0.769, and 0.739 respectively], whereas the discrimination ability of SIRS for in-hospital mortality was poor (AUROC = 0.595) (Figure 2). The addition of the FI-Lab to each score significantly improved its ability to identify those who died in the hospital (all p for Δ C-statistic < 0.001; Table 4; Figure 2). When the FI-Lab was included, the discrimination abilities of all scores were improved, with an IDI of 0.056, 0.026, 0.050, 0.040, 0.067, and 0.129 for SOFA, APS3, SAPS2, LODS, and OASIS, and SIRS, respectively (Table 4). The net improvement in predicted probabilities increased significantly (NRI = 0.505, 0.350, 0.495, 0.491, 0.570, and 0.723 for SOFA, APS 3, SAPS 2, LODS, OASIS, and SIRS, respectively; Table 4).

**TABLE 4:** Incremental value of frailty index based on laboratory tests (FI-Lab) for in-hospital and 1-year mortality.

	C-statistic^a^	C-statistic^b^	Δ C-statistic	*P*-value for Δ C	IDI (95% CI)	NRI (95% CI)
**In-hospital mortality**						
SOFA **+** FI-Lab vs. SOFA	0.765 (0.741–0.789)	0.716 (0.689–0.742)	0.049	<0.001	0.056 (0.045–0.067)	0.505 (0.407–0.603)
APS3 **+** FI-Lab vs. APS3	0.803 (0.781–0.825)	0.789 (0.766–0.811)	0.014	0.006	0.026 (0.018–0.034)	0.350 (0.250–0.450)
SAPS2 **+** FI-Lab vs. SAPS2	0.781 (0.759–0.804)	0.746 (0.722–0.771)	0.035	<0.001	0.050 (0.040–0.061)	0.495 (0.396–0.593)
LODS **+** FI-Lab vs. LODS	0.796 (0.773–0.819)	0.769 (0.744–0.793)	0.027	<0.001	0.040 (0.030–0.050)	0.491 (0.392–0.589)
OASIS **+** FI-Lab vs. OASIS	0.788 (0.765–0.810)	0.739 (0.714–0.764)	0.049	<0.001	0.067 (0.055–0.079)	0.570 (0.473–0.667)
SIRS **+** FI-Lab vs. SIRS	0.752 (0.728–0.776)	0.595 (0.567–0.623)	0.157	<0.001	0.129 (0.112–0.145)	0.723 (0.629–0.817)
**1-year mortality**						
SOFA **+** FI-Lab vs. SOFA	0.694 (0.671–0.715)	0.636 (0.612–0.660)	0.058	<0.001	0.057 (0.047–0.066)	0.413 (0.329–0.496)
APS3 **+** FI-Lab vs. APS3	0.730 (0.709–0.751)	0.723 (0.701–0.744)	0.007	0.152	0.019 (0.013–0.025)	0.269 (0.184–0.354)
SAPS2 **+** FI-Lab vs. SAPS2	0.734 (0.711–0.753)	0.720 (0.699–0.741)	0.014	0.023	0.028 (0.021–0.035)	0.302 (0.217–0.386)
LODS **+** FI-Lab vs. LODS	0.721 (0.701–0.744)	0.696 (0.674–0.719)	0.025	<0.001	0.032 (0.025–0.040)	0.350 (0.265–0.434)
OASIS **+** FI-Lab vs. OASIS	0.713 (0.691–0.734)	0.668 (0.645–0.691)	0.045	<0.001	0.053 (0.043–0.063)	0.408 (0.325–0.492)
SIRS **+** FI-Lab vs. SIRS	0.691 (0.666–0.711)	0.548 (0.523–0.572)	0.143	<0.001	0.103 (0.090–0.116)	0.535 (0.452–0.617)

^a^Models including disease severity score and FI-Lab.

^b^Models only including disease severity score.

FI-Lab, frailty index based on laboratory tests; SOFA, sequential organ failure assessment; APS, acute physiology score; SAPS, simplified acute physiology score; LODS, logistic organ dysfunction score; OASIS, Oxford acute severity of illness score; SIRS, systemic inflammatory response syndrome.

**FIGURE 3:**
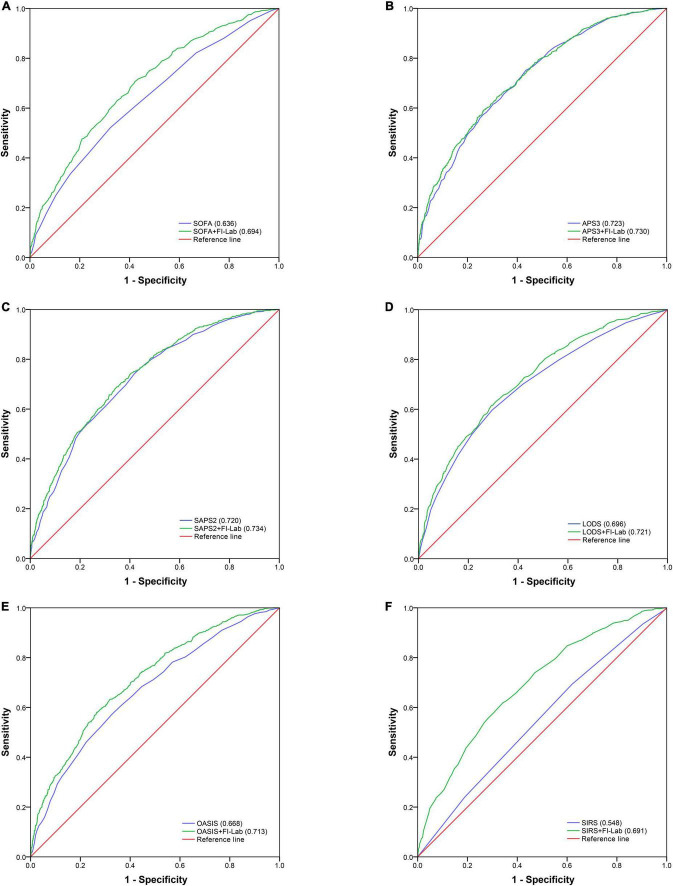
Area under the receiver operator curve for 1-year mortality.

Acute physiology score (APS) 3 and SAPS 2 had good discrimination abilities for 1-year mortality (AUROC = 0.723 and 0.720, respectively); SOFA, LODS, and OASIS had moderate discrimination abilities (AUROC = 0.636, 0.696, and 0.668, respectively); and SIRS had poor discrimination ability (AUROC = 0.548) (Figure 3). The addition of the FI-Lab improved the abilities of SOFA, SAPS2, LODS, OASIS, and SIRS, but not that of APS 3, to identify those who died within 1 year (Table 4; Figure 3). When the FI-Lab was included, the discrimination for all scores improved significantly, and the net improvement for all scores in predicted probabilities also increased significantly (Table 4).

## Discussion

To our knowledge, this study is the first to explore the association between the FI-Lab and short-and long-term mortality, as well as the incremental value of the FI-Lab in risk stratification in patients with critical AMI. Our results showed that the FI-Lab was independently associated with short-and long-term mortality in critical AMI patients; the FI-Lab had incremental value for mortality when added to classic disease severity scores.

The FI-Lab has recently been expanded from epidemiological studies to clinical practice because it can identify a high-risk subgroup of patients with a poor prognosis. Ritt et al. found that the FI-Lab was associated with increased 6-months and 1-year mortality in patients hospitalized in geriatric ward (12). A study by Kim et al. showed that higher FI-Lab scores were associated with longer hospital length of stay (LOS), increased readmission within 30 days of surgery, ICU admission, and increased mortality in older surgical patients with cancer (11). The FI-Lab also had prognostic value for patients with cardiovascular disease. Lim’s study found that a high preoperative FI-Lab score was associated with a higher risk of adverse postoperative outcomes, including ICU, hospital LOS, and readmission within 30 days, among coronary artery bypass graft surgery patients (18). Overall, clinical evidence of the application of the FI-Lab in inpatients, including patients with AMI, is still lacking; thus, further research is warranted.

Frailty captures most of the prognostic information provided by geriatric conditions and may also predict outcomes beyond age and standard risk factors; it can thus add incremental value for traditional risk stratification tools. The present study first showed that the FI-Lab increased the ability of classic disease severity scores to predict short-and long-term mortality in critical AMI patients in an ICU setting. For the risk prediction of patients with STEMI, Matsuzawa et al. found that the addition of gait speed as a measure of frailty to the Framingham risk score improved reclassification (NRI: 32.8%, *p* < 0.001) of cardiovascular events (cardiovascular deaths, non-fatal myocardial infarctions, and non-fatal ischemic strokes) (19). Sanchis et al. found that the addition of frailty, as assessed by the Green score ≥ 5, to the Global Registry of Acute Coronary Events (GRACE) score improved discrimination (AUROC: 0.776 vs. 0.726, *p* < 0.001) and significantly reclassified the mortality risk (NRI: 50.5%, *p* < 0.001) of patients diagnosed with acute coronary syndrome (ACS) (6). Furthermore, Campo et al. demonstrated that scales of frailty and physical performance can enhance the likelihood [*via* the GRACE and Thrombolysis in Myocardial Infarction (TIMI) scores] of predicting negative prognoses in older adults after ACS (20). Anand’s study arrived at a similar result, finding that frailty as assessed by the CFS enhanced the discrimination of GRACE for 12 months for all-cause mortality (AUROC: 0.86 vs. 0.80, *p* = 0.04) in older patients with AMI (21).

There are several important issues to consider when using the FI-Lab. First, the FI-Lab could only reflect the frailty status over a short time before and after the measurement of blood tests. It may thus be highly variable during residency and if there are changes in the disease severity. Thus, other FI-Lab values, such as mean FI-Lab of residency and FI-Lab before discharge, can also be calculated. It remains unclear which of the above FI-Lab values has the best predictive ability for adverse outcomes. Second, the optimal number of items to construct the FI-Lab remains unknown. Howlett et al. used 23 items to calculate the FI-Lab, whereas the present study utilized 33 items for the construction of the FI-Lab. The minimum and maximum number of items for FI-Lab construction needs to be explored in the future. Third, the original study constructed the FI-Lab using common laboratory tests of blood samples and vital signs (blood pressure), whereas we additionally applied several items from arterial blood gas samples and urine samples to construct our FI-Lab. Ritt et al. also operationalized the FI-Lab using routine blood and urine tests (12). The method for choosing the appropriate laboratory tests from various samples (blood, blood gas, and urine) and the representative biological systems or organs is not yet clear. Fourth, there is no consensus cut-off value of the FI-Lab for pre-frailty or frailty. Several previous studies have referred to the cut-off values of the FI developed by Rockwood et al. (22). However, this might not be appropriate because the distribution the FI-Lab and FI are different even in the same population. Finally, a majority of older adults and patients took multiple drugs to control comorbidities, such as hypertension, diabetes, anemia, dyslipidemia, and vitamin deficiency. As such, their normal levels of blood pressure, glucose, hemoglobin, cholesterol, and vitamins come from therapeutic drugs, making their corresponding FI-Lab underestimated. Future research is needed to explore the aforementioned issues.

This study has several strengths. First, this study had a large sample size and used high-quality data from the MIMIC-IV database. Therefore, the results and conclusion of our study are credible. Second, to the best of our knowledge, this is the first study to demonstrate the prognostic role of the FI-Lab in critical AMI patients. We also demonstrated the incremental value of the FI-Lab for mortality over several classic disease severity scores. However, the following limitations exist. We only included critical AMI patients, thus the results of this study cannot be generally applied to all AMI patients. Furthermore, the GRACE and TIMI scores were significantly associated with mortality in patients with AMI. Owing to the lack of GRACE and TIMI information in the MIMIC-IV database, we did not include these scores in the multivariable model. As such, we could not investigate the incremental value of the FI-Lab over these two scores. Lastly, the present study was a single-center study.

## Conclusion

The current study showed that the FI-Lab is a strong predictor of short-and long-term mortality in critical AMI patients. The FI-Lab might enhance the ability to predict negative outcomes in patients with critical AMI and could improve the clinical evaluation of such patients and support a tailored decision-making process regarding their care.

## Data availability statement

Publicly available datasets were analyzed in this study. This data can be found here: https://physionet.org.

## Ethics statement

The studies involving human participants were reviewed and approved by Institutional Review Boards (IRB) of the Massachusetts Institute of Technology. The patients/participants provided their written informed consent to participate in this study.

## Author contributions

WX and LQ designed, supervised the study, and critically revised the manuscript. WB and BH curated, harmonized the data, and drafted the manuscript. WB and LX performed the statistical analyses. WB, WX, and JQ critically reviewed all statistical methods, procedures, and results. All authors listed have made a substantial, direct, and intellectual contribution to the work, and approved it for publication.
