# Supplementary material for: Frailty index based on laboratory tests improves prediction of short-and long-term mortality in patients with critical acute myocardial infarction
Source: Front Med (Lausanne). 2022 Dec 6;9:1070951. doi: 10.3389/fmed.2022.1070951 (PMC9763273; doi:10.3389/fmed.2022.1070951)

**Table S1. Reference range of items used for construction of FI-Lab**

| Items | Reference range |
| --- | --- |
| Vital signs |  |
| Systolic blood pressure (mm Hg) | 90–140 |
| Diastolic blood pressure (mm Hg) | 60–90 |
| Heart rate (bpm) | 60–99 |
| Venous blood samples |  |
| White cell count (×10^3^/μL) | 4–11 |
| Platelet count (×10^9^/L) | 150–440 |
| Hemoglobin (g/dL) | Female: 12–16 Male: 14–18 |
| Total bilirubin (mg/dL) | 0–1.5 |
| Alanine transaminase (Units/L) | 0–40 |
| Albumin (g/dL) | 3.5–5 |
| Alkaline phosphatase (Units/L) | 35–105 |
| Lactate dehydrogenase (Units/L) | 94–250 |
| Urea nitrogen (mg/dL) | 6–20 |
| Creatinine (mg/dL) | Female: 0.4–1.1 Male: 3.5–5.2 |
| Glucose (mg/dL) | 70–110 |
| Potassium (mmol/L) | 3.5–5.4 |
| Sodium (mmol/L) | 133–145 |
| Calcium (mg/dL) | 8.4–10.3 |
| Phosphorus (mg/dL) | 2.7–4.5 |
| Prothrombin time (s) | 9.4–12.5 |
| International normalized ratio | 0.9–1.1 |
| APTT (s) | 25–35 |
| Fibrinogen (mg/dL) | 150–400 |
| Troponin T (ng/mL) | 0–0.01 |
| Arterial blood gas samples |  |
| PH | 7.35–7.45 |
| PO_2_ (mm Hg) | 85–105 |
| PCO_2_ (mm Hg) | 35–45 |
| Lactate (mmol/L) | 0.5–2 |
| Urine sample |  |
| Leucocytes | Negative |
| Erythrocytes | Negative |
| Protein | Negative |
| Glucose | Negative |
| Ketones | Negative |
| Bilirubin | Negative |

APTT, activated partial thromboplastin time; PH, potential of hydrogen; PO2, partial pressure of oxygen; PCO2, partial pressure of carbon dioxide; SD, standard deviation.

**Table S2. Detailed scoring rules of sequential organ failure assessment score (SOFA score)**

|  | Central nervous  System | Cardiovascular  System | Respiratory  System | Coagulation | Liver | Renal function |
| --- | --- | --- | --- | --- | --- | --- |
| Score | Glasgow coma scale | Mean arterial pressure OR administration of vasopressors | PaO_2_/FiO_2_  [mmHg (kPa)] | Platelets*10^3^/ul | Bilirubin (mg/dl)  [µmol/L] | Creatinine (mg/dl) [µmol/L]  (or urine output) |
| 0 | 15 | MAP ≥ 70 mmHg | ≥ 400 (53.3) | ≥ 150 | < 1.2 [<20] | <1.2 [<110] |
| 1 | 13-14 | MAP < 70 mmHg | < 400 (53.3) | <150 | 1.2-1.9 [20-32] | 1.2-1.9 [110-170] |
| 2 | 10-12 | dopamine ≤ 5 µg/kg/min or dobutamine (any dose) | < 300 (40) | <100 | 2.0-5.9 [33-101] | 2.0-3.4 [171-299] |
| 3 | 6-9 | dopamine > 5µg/kg/min OR epinephrine ≤ 0.1µg/kg/min OR norepinephrine ≤ 0.1µg/kg/min | < 200 (26.7) and mechanically ventilated including CPAP | <50 | 6.0-11.9 [102-204] | 3.5-4.9 [300-400]  (or < 500ml/day) |
| 4 | <6 | dopamine > 15 μg/kg/min OR epinephrine > 0.1 μg/kg/min OR norepinephrine > 0.1 μg/kg/min | < 100 (13.3) and mechanically ventilated including CPAP | <20 | >12.0 [>204] | >5.0 [>440]  (or < 200ml/day) |

**Table S3. Detailed scoring rules of simplified acute physiology score (SAPS) II**

| Varibales | Score | | | | | | | | | | | | | | | | | | | | |
| --- | --- | --- | --- | --- | --- | --- | --- | --- | --- | --- | --- | --- | --- | --- | --- | --- | --- | --- | --- | --- | --- |
|  | 26 | 13 | 12 | 11 | 9 | 7 | 6 | 5 | 4 | 3 | 2 | 0 | 1 | 2 | 3 | 4 | 5 | 6 | 7 | 9 | 10 |
| HR (beats/min) |  |  |  | <40 |  |  |  |  |  |  | 40-69 | 70-119 |  |  |  | 120-159 |  |  | ≥ 160 |  |  |
| SBP (mmHg) |  | <70 |  |  |  |  |  | 70-99 |  |  |  | 100-199 |  | ≥200 |  |  |  |  |  |  |  |
| Temperature (℃) |  |  |  |  |  |  |  |  |  |  |  | <39 |  |  | ≥ 39 |  |  |  |  |  |  |
| PaO_2_/FiO_2_ only if VENT or CPAP |  |  |  | <100 | 100-199 | ≥200 |  |  |  |  |  |  |  |  |  |  |  |  |  |  |  |
| Urine output (L/day) |  |  |  | <0.5 |  |  |  |  | 0.5-1 |  |  | ≥1 |  |  |  |  |  |  |  |  |  |
| Urea (g.L) |  |  |  |  |  |  |  |  |  |  |  | <0.6 |  |  |  |  |  | 0.6-1.7 |  |  | ≥1.8 |
| TLC |  |  | <1 |  |  |  |  |  |  |  |  | 1-19.9 |  | ≥20 |  |  |  |  |  |  |  |
| Potassium |  |  |  |  |  |  |  |  |  | <3 |  | 3-4.9 |  | ≥5 |  |  |  |  |  |  |  |
| Sodium |  |  |  |  |  |  |  | <125 |  |  |  | 125-144 | ≥145 |  |  |  |  |  |  |  |  |
| Bicarbonate |  |  |  |  |  |  | <15 |  |  | 15-19 |  | ≥20 |  |  |  |  |  |  |  |  |  |
| Bilirubin (mg/dl) |  |  |  |  |  |  |  |  |  |  |  | <40 |  |  |  | 40-59.9 |  |  |  | ≥60 |  |
| GCS | <6 | 6-8 |  |  |  | 9-10 |  | 11-13 |  |  |  | 14-15 |  |  |  |  |  |  |  |  |  |
| Age | | | | Score | | | Chronic disease | | | | Score | | | Type of admission | | | | Score | | | |
| <40 | | | | 0 | | | Metastatic cancer | | | | 9 | | | Scheduled surgical | | | | 0 | | | |
| 40-59 | | | | 7 | | | Hematological malignancy | | | | 10 | | | Medical | | | | 6 | | | |
| 60-69 | | | | 12 | | | AIDS | | | | 17 | | | Emergency surgical | | | | 8 | | | |
| 70-74 | | | | 15 | | |  | | | |  | | |  | | | |  | | | |
| 75-79 | | | | 16 | | |  | | | |  | | |  | | | |  | | | |
| >80 | | | | 18 | | |  | | | |  | | |  | | | |  | | | |
| SAPS II score | | | |  | | | | | | | | | | | | | | | | | |

**Table S4. Detailed scoring rules of acute physiology score (APS) III**

| Demographics/Previous health status | | Diagnostic category | | Physiologic parameters on admission | |
| --- | --- | --- | --- | --- | --- |
| Parameters | Score | Parameters | Score | Parameters | Score |
| Age |  | Scheduled admission | 0 | Glasgow |  |
| < 40 | 0 | Non-scheduled admission | 3 | 3-4 | 15 |
| 40-59 | 5 | Urgency |  | 5 | 10 |
| 60-69 | 9 | Non-surgical | 5 | 6 | 7 |
| 70-74 | 13 | Elective | 0 | 7-12 | 2 |
| 75-79 | 15 | Emergency | 6 | ≥ 13 | 0 |
| ≥ 80 | 18 | Type of surgery |  | Heart rate |  |
| Comorbidities |  | Transplantation | -11 | <120 | 0 |
| Others | 0 | Trauma | -8 | 120-159 | 5 |
| Chemotherapy | 3 | MR without valve | -6 | ≥160 | 7 |
| ICC NYHA IV | 6 | Stroke surgery | 5 | Systolic blood pressure |  |
| Hematologic Neoplasia | 6 | Other | 0 | <40 | 11 |
| Cirrhosis | 8 | ICU admission add 16 points | 16 | 40-69 | 8 |
| Aids | 8 | Reason for admission |  | 70-119 | 3 |
| Metastasis | 11 | Neirologic |  | ≥ 120 | 0 |
| In-hospital days before ICU |  | Seizures | -4 | Oxygenation |  |
| <14 | 0 | Coma, confusion, agitation | 4 | MV PaO_2_/FiO_2_<100 | 11 |
| 14-27 | 6 | Focal deficit | 7 | MV PaO_2_/FiO_2_≥100 | 7 |
| ≥28 | 7 | Intracranial mass effect | 11 | Without MV PaO_2_ <60 | 5 |
| Origin |  | Cardiologic |  | Without MV PaO_2_ ≥60 | 0 |
| Operating room | 0 | Arrhythmia | -5 | Temperature |  |
| ER | 5 | Hemorrhagic shock | 3 | <34.5 | 7 |
| Other ICU | 7 | Non-hemorrhagic hypovolemic shock | 3 | ≥34.5 | 0 |
| Others | 8 | Distributive shock | 5 | Leukocytes |  |
| Vasoactive drugs |  | Abdomen |  | <15000 | 0 |
| Yes | 0 | Acute abdomen | 3 | ≥15000 | 2 |
| No | 3 | Severe pancreatitis | 9 | Platelets |  |
|  |  | Liver failure | 6 | <20000 | 13 |
|  |  | Others | 0 | 20000-49999 | 8 |
|  |  | Infection |  | 50000-99999 | 5 |
|  |  | Nosocomial | 4 | ≥100000 | 0 |
|  |  | Respiratory | 5 | pH |  |
|  |  | Others | 0 | ≤7.25 | 3 |
|  |  |  |  | >7.25 | 0 |
|  |  |  |  | Creatinine |  |
|  |  |  |  | <1.2 | 0 |
|  |  |  |  | 1.2-1.9 | 2 |
|  |  |  |  | 2.0-3.4 | 7 |
|  |  |  |  | ≥3.5 | 8 |
|  |  |  |  | Bilirubin |  |
|  |  |  |  | <2 | 0 |
|  |  |  |  | 2-5.9 | 4 |
|  |  |  |  | ≥6 | 5 |
| Total |  |  |  |  |  |

**Table S5. Detailed scoring rules of logistic organ dysfunction score (LODS)**

| Measurements of organic systems | 5 | 3 | 1 | 0 | 1 | 3 | 5 |
| --- | --- | --- | --- | --- | --- | --- | --- |
| Neurological (GCS) | 3-5 | 6-8 | 9-13 | 14-15 |  |  |  |
| Cardiovascular |  |  |  |  |  |  |  |
| HR (beats/min) | <30 | 40-69 | 70-89 | 30-139 | ≥ 140 |  |  |
| SBP (mmHg) | <40 |  |  | 0-239 | 240-269 | ≥ 270 |  |
| Renal |  |  |  |  |  |  |  |
| Ureic nitrogen (mmol/L) |  |  |  | <6 | 6-9.98 | 9.99-19.98 | ≥19.99 |
| Serum creatinine (µmol/L) |  |  |  | <106.08 | 106.08-140.55 | ≥141.44 |  |
| Urine output (L/24h) | <0.5 | 0.5-0.74 |  | 0.75-0.99 |  | ≥10 |  |
| Respiratory |  |  |  |  |  |  |  |
| PaO_2_/FiO_2_ in MV or CPAP |  | <150 | ≥150 | With no ventialtion, CPAP or IPAP |  |  |  |
| Hematologic |  |  |  |  |  |  |  |
| TLC (mm^3^)*10^3^ |  | <1.0 | 1.0-2.4 | 2.5-49.9 | ≥50 |  |  |
| Platelets (mm^3^)*10^3^ |  |  | <50 | ≥50 |  |  |  |
| Hepatic |  |  |  |  |  |  |  |
| Serum bilirubin (µmol/L) |  |  |  | <34.2 | ≥34.2 |  |  |
| PT (seconds and %) |  |  | <25 | <3s, >25 | ≥ 3s |  |  |

**Table S6. Detailed scoring rules of Oxford Acute Severity of Illness Score (OASIS)**

| Critierion | Range | score |
| --- | --- | --- |
| Pre-ICU Length of stay (hours) | <0.17 | 5 |
|  | 0.17-4.94 | 3 |
|  | 4.95-24.00 | 0 |
|  | 24.01-311.80 | 2 |
|  | >311.80 | 1 |
| Age | <24 | 0 |
|  | 24-53 | 3 |
|  | 54-77 | 6 |
|  | 77-89 | 9 |
|  | >90 | 7 |
| Heart rate (beats/min) | <33 | 4 |
|  | 33-88 | 0 |
|  | 89-106 | 1 |
|  | 107-125 | 3 |
|  | >125 | 6 |
| MAP (mmHg) | <20.65 | 4 |
|  | 20.65-50.99 | 3 |
|  | 51.00-61.32 | 2 |
|  | 62.33-143.44 | 0 |
|  | >143.44 | 3 |
| Respiratory rate (breaths/min) | <6 | 10 |
|  | 6-12 | 1 |
|  | 13-22 | 0 |
|  | 23-30 | 1 |
|  | 31-44 | 6 |
|  | >44 | 9 |
| Temperature (℃) | <33.22 | 3 |
|  | 33.22-35.93 | 4 |
|  | 35.94-36.39 | 2 |
|  | 36.40-36.88 | 0 |
|  | 36.89-39.88 | 2 |
|  | >39.88 | 6 |
| Urine output (ml) | <671 | 10 |
|  | 671-1426.99 | 5 |
|  | 1427-2543.99 | 1 |
|  | 2544-6896 | 0 |
|  | >6896 | 8 |
| Ventilated | Yes | 9 |
|  | No | 0 |
| Elective surgery | Yes | 6 |
|  | No | 0 |

**Table S7. Detailed scoring rules of systemic inflammatory response synfrome (SIRS) score**

| Criterion | Threshold | Score |
| --- | --- | --- |
| Body temperatire (℃) | <36 or >38 | 1 |
| Heart rate (beats/min) | >90 | 1 |
| White blood cell count (10^3^/L) | <4 or >12 | 1 |
| Respiratory rate (breaths/min) | >22 | 1 |

**Table S8. Baseline characteristics of included and excluded patients**

| Variable | Included patients  N=2159 | Excluded patients  N=2844 | *P* value |
| --- | --- | --- | --- |
| Age (year) | 72 (62-81) | 73 (61-81) | 0.254 |
| Men (n, %) | 1298 (60.1) | 1763 (65.8) | 0.179 |
| Ethnicity (n, %) |  |  | 0.586 |
| White | 1372 (63.5) | 1777 (62.5) |  |
| Black | 173 (8.0) | 249 (8.8) |  |
| Other | 614 (28.4) | 818 (28.8) |  |
| STEMI (n, %) | 744 (34.5) | 1097 (36.0) | 0.268 |
| Troponin T (ng/mL) | 0.40 (0.13-1.39) | 0.48 (0.16-1.59) | <0.001 |
| FI-Lab (score) | 0.45 (0.36-0.55) | 0.46 (0.36-0.57) | 0.165 |
| Disease severity scoring system (score) |  |  |  |
| SOFA | 6 (3-8) | 6 (2-8) | 0.254 |
| APS 3 | 52 (39-72) | 50 (36-70) | 0.091 |
| SAPS 2 | 41 (33-51) | 39 (31-51) | 0.142 |
| LODS | 6 (4-9) | 6 (4-9) | 0.322 |
| OASIS | 36 (29-43) | 34 (28-44) | 0.102 |
| SIRS | 3 (2-3) | 3 (2-3) | 0.511 |

**Table S9. Univariate association between each items included in the FI-Lab and in-hospital mortality**

| Items | In-hospital mortality | C-statistic |
| --- | --- | --- |
| Vital signs |  |  |
| Systolic blood pressure (mm Hg) | 1.17 (0.83-1.65) | 0.506 (0.477-0.536) |
| Diastolic blood pressure (mm Hg) | 1.33 (1.08-1.63) | 0.535 (0.516-0.565) |
| Heart rate (bpm) | 1.98 (1.57-2.50) | 0.560 (0.530-0.590) |
| Venous blood samples |  |  |
| White cell count (×10^3^/μL) | 1.87 (1.49-2.35) | 0.567 (0.539-0.595) |
| Platelet count (×10^9^/L) | 1.55 (1.25-1.92) | 0.549 (0.519-0.579) |
| Hemoglobin (g/dL) | 1.19 (0.87-1.61) | 0.510 (0.481-0.539) |
| Total bilirubin (mg/dL) | 2.77 (2.07-3.72) | 0.554 (0.524-0.585) |
| Alanine transaminase (Units/L) | 2.30 (1.87-2.83) | 0.600 (0.571-0.629) |
| Albumin (g/dL) | 1.99 (1.62-2.44) | 0.584 (0.555-0.613) |
| Alkaline phosphatase (Units/L) | 2.21 (1.78-2.74) | 0.583 (0.553-0.613) |
| Lactate dehydrogenase (Units/L) | 2.19 (1.78-2.70) | 0.597 (0.568-0.626) |
| Urea nitrogen (mg/dL) | 2.88 (2.22-3.75) | 0.598 (0.571-0.626) |
| Creatinine (mg/dL) | 1.46 (1.12-1.89) | 0.531 (0.502-0.560) |
| Glucose (mg/dL) | 1.39 (1.03-1.89) | 0.520 (0.491-0.549) |
| Potassium (mmol/L) | 1.72 (1.28-2.30) | 0.530 (0.500-0.560) |
| Sodium (mmol/L) | 2.05 (1.59-2.65) | 0.552 (0.522-0.583) |
| Calcium (mg/dL) | 1.77 (1.43-2.18) | 0.569 (0.540-0.598) |
| Phosphorus (mg/dL) | 1.90 (1.55-2.34) | 0.577 (0.548-0.607) |
| Prothrombin time (s) | 2.35 (1.80-3.08) | 0.573 (0.545-0.601) |
| International normalized ratio | 2.54 (1.71-3.77) | 0.541 (0.513-0.570) |
| APTT (s) | 1.35 (1.09-1.68) | 0.534 (0.505-0.563) |
| Fibrinogen (mg/dL) | 2.36 (1.79-3.12) | 0.552 (0.522-0.583) |
| Troponin T (ng/mL) | 2.18 (1.52-3.13) | 0.539 (0.511-0.567) |
| Arterial blood gas samples |  |  |
| PH | 2.74 (2.21-3.39) | 0.623 (0.595-0.651) |
| PO_2_ (mm Hg) | 1.28 (1.01-1.64) | 0.523 (0.494-0.552) |
| PCO_2_ (mm Hg) | 1.48 (1.20-1.81) | 0.547 (0.518-0.577) |
| Lactate (mmol/L) | 3.12 (2.51-3.86) | 0.637 (0.610-0.665) |
| Urine sample |  |  |
| Leucocytes | 1.85 (1.50-2.27) | 0.576 (0.547-0.605) |
| Erythrocytes | 1.66 (1.35-2.05) | 0.562 (0.533-0.591) |
| Protein | 1.60 (1.22-2.10) | 0.536 (0.507-0.565) |
| Glucose | 1.03 (0.82-1.30) | 0.503 (0.474-0.533) |
| Ketones | 1.12 (0.89-1.40) | 0.511 (0.482-0.541) |
| Bilirubin | 2.37 (1.71-3.29) | 0.537 (0.507-0.567) |

APTT, activated partial thromboplastin time; PH, potential of hydrogen; PO2, partial pressure of oxygen; PCO2, partial pressure of carbon dioxide; SD, standard deviation.

**Table S10. Univariate association between each items included in the FI-Lab and 1-year mortality**

| Items | 1-year mortality | C-statistic |
| --- | --- | --- |
| Vital signs |  |  |
| Systolic blood pressure (mm Hg) | 1.14 (0.85-1.54) | 0.505 (0.481-0.530) |
| Diastolic blood pressure (mm Hg) | 1.26 (1.06-1.49) | 0.529 (0.504-0.553) |
| Heart rate (bpm) | 1.45 (1.17-1.79) | 0.530 (0.505-0.555) |
| Venous blood samples |  |  |
| White cell count (×10^3^/μL) | 1.35 (1.13-1.62) | 0.534 (0.510-0.559) |
| Platelet count (×10^9^/L) | 1.40 (1.16-1.68) | 0.536 (0.512-0.561) |
| Hemoglobin (g/dL) | 1.36 (1.06-1.76) | 0.518 (0.493-0.542) |
| Total bilirubin (mg/dL) | 2.10 (1.58-2.80) | 0.534 (0.509-0.559) |
| Alanine transaminase (Units/L) | 1.52 (1.27-1.81) | 0.549 (0.524-0.574) |
| Albumin (g/dL) | 1.95 (1.64-2.33) | 0.580 (0.556-0.605) |
| Alkaline phosphatase (Units/L) | 2.04 (1.68-2.48) | 0.570 (0.545-0.594) |
| Lactate dehydrogenase (Units/L) | 1.57 (1.32-1.86) | 0.555 (0.531-0.580) |
| Urea nitrogen (mg/dL) | 3.28 (2.67-4.02) | 0.619 (0.595-0.642) |
| Creatinine (mg/dL) | 1.10 (0.89-1.35) | 0.508 (0.483-0.533) |
| Glucose (mg/dL) | 1.22 (0.96-1.55) | 0.513 (0.488-0.537) |
| Potassium (mmol/L) | 1.67 (1.28-2.19) | 0.526 (0.501-0.551) |
| Sodium (mmol/L) | 1.93 (1.52-2.44) | 0.543 (0.518-0.568) |
| Calcium (mg/dL) | 1.39 (1.16-1.64) | 0.540 (0.515-0.564) |
| Phosphorus (mg/dL) | 1.87 (1.56-2.23) | 0.573 (0.548-0.597) |
| Prothrombin time (s) | 1.84 (1.50-2.25) | 0.558 (0.534-0.582) |
| International normalized ratio | 1.73 (1.32-2.27) | 0.529 (0.504-0.553) |
| APTT (s) | 1.20 (1.00-1.44) | 0.521 (0.496-0.546) |
| Fibrinogen (mg/dL) | 1.82 (1.40-2.37) | 0.532 (0.507-0.557) |
| Troponin T (ng/mL) | 2.72 (2.05-3.62) | 0.553 (0.529-0.578) |
| Arterial blood gas samples |  |  |
| PH | 1.93 (1.62-2.30) | 0.581 (0.557-0.606) |
| PO_2_ (mm Hg) | 1.08 (0.89-1.31) | 0.507 (0.482-0.532) |
| PCO_2_ (mm Hg) | 1.36 (1.14-1.62) | 0.537 (0.512-0.561) |
| Lactate (mmol/L) | 1.94 (1.63-2.31) | 0.582 (0.558-0.607) |
| Urine sample |  |  |
| Leucocytes | 2.11 (1.78-2.51) | 0.592 (0.568-0.617) |
| Erythrocytes | 1.55 (1.31-1.85) | 0.554 (0.530-0.579) |
| Protein | 2.03 (1.63-2.54) | 0.556 (0.532-0.580) |
| Glucose | 0.90 (0.75-1.10) | 0.510 (0.485-0.535) |
| Ketones | 0.92 (0.76-1.12) | 0.508 (0.483-0.533) |
| Bilirubin | 2.25 (1.63-3.10) | 0.530 (0.505-0.555) |

APTT, activated partial thromboplastin time; PH, potential of hydrogen; PO2, partial pressure of oxygen; PCO2, partial pressure of carbon dioxide; SD, standard deviatio

**Figure S1. Detailed patients selection process.**


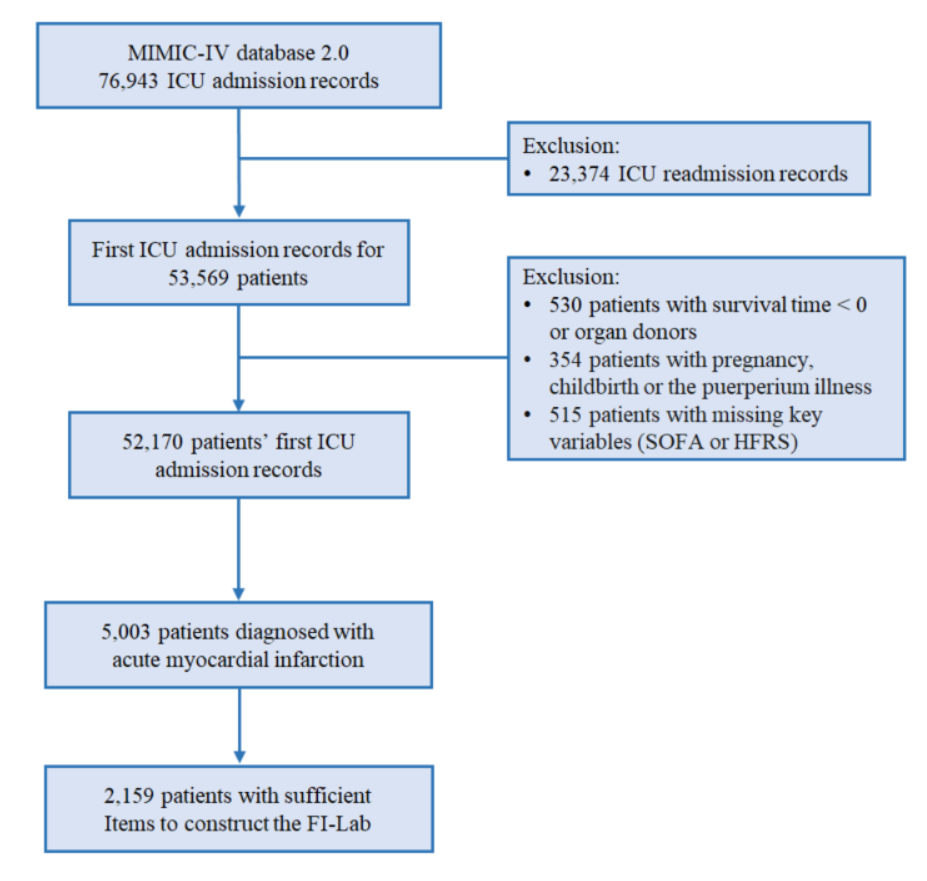

Supplement: Supplementary file 1 [file Data_Sheet_1.docx]
